# Spontaneous rotation of a toric implantable collamer lens related to abnormal ciliary body morphology: a case report

**DOI:** 10.1186/s12886-020-01597-5

**Published:** 2020-08-28

**Authors:** Qian Chen, Qingyan Zeng, Zheng Wang, Chao Pan, Xiaohua Lei, Weina Tan

**Affiliations:** 1 Aier Eye Hospital of Wuhan University, Wuhan, Hubei Province China; 2 Hankou Aier Eye Hospital, Machang Road, Jianghan District, Wuhan, Hubei Province China; 3 Aier Institute of Refractive Surgery, Guangzhou, Guangdong Province China

**Keywords:** Ciliary body, Myopic astigmatism, Rotation, Toric implantable Collamer Lens

## Abstract

**Background:**

This is a case of spontaneous toric implantable collamer lens (TICL) rotation that occurred twice in the left eye of a patient.

**Case presentation:**

A 24-year-old gentleman received TICL implantation for treatment of myopic astigmatism encountered with spontaneous rotation of approximately 90° from its original position. TICL reposition procedure was performed with visual outcome of 20/16. Surprisingly, a re-rotation of TICL occurred. The patient underwent a replacement of non-toric ICL with a larger size after careful re-evaluation with final visual outcome of 20/20. A short and small ciliary process with shallow ciliary sulcus and posteriorly positioned ciliary body was found by Ultrasound Biomicroscopy (UBM).

**Conclusions:**

The unique morphology of the ciliary body may have a potential association with the vault and instability of implanted ICL. Careful examination of the ciliary body morphology is essential in preoperative evaluation.

## Background

The Visian Toric Implantable Collamer Lens (TICL) (STAAR Surgical, Nidau, Switzerland) is a foldable lens designed to correct myopia and myopic astigmatism. Previous studies have revealed that the position of the V4C TICL in the eye is stable with an average rotation angle of 3.39 ± 2.36° [1]. According to the current literature, there is only few reports of spontaneous TICL rotation that led significant decrease in visual acuity and required further surgical procedures [2, 3].Here, we present a rare case in which a TICL spontaneously rotated approximately 90° twice and analyze the possible causes of the rotation and the finally successful management strategy.

## Case presentation

A 24-year-old gentleman with no history of systemic and ocular diseases and surgery presented for refractive surgery. The patient’s uncorrected distance visual acuity (UDVA) was 20/160 in the right eye and 20/200 in the left eye, with refractive error of − 7.00 -1.00 × 5° and - 9.50 - 2.00 × 175°. The anterior segment and fundus evaluations were unremarkable. Keratometric values, central corneal thickness, anterior chamber depth from the endothelium of the cornea in bilateral eyes measured by Pentacam HR (Oculus, Germany) were 43.3@96/42.1@6 and 43.8@93/42.3@3, 574 μm and 571 μm, 3.27 mm and 3.20 mm, respectively, while white-to-white distance obtained with a caliper were both 10.93 mm, horizontal sulcus-to-sulcus distance assessed by the UBM (Model SW-3200 L;Tianjin Suowei Electonic Technology Co, Ltd., Tianjin, China) were 11.28 mm and 11.16 mm.

After a thorough discussion with the patient, regarding the risks and benefits of surgery, an informed consent was obtained. The right eye undergone a LASIK procedure, while in the left eye TICL calculation software developed by the STAAR Surgical company recommended V4C (model: TICM12.1) with a power of - 12.50 + 2.0 × 91° and 6° clockwise rotation after horizontal implantation 7 days later. Zero and 180 degree limbus reference marking was identified under the slit-lamp with patient sitting upright preoperatively. After topical anesthesia, an inferior paracentesis and 3.0 mm temporal clear corneal incision were made. The TICL was inserted through the temporal incision with four haptics tucked behind the iris and subsequently axis adjusted to the desired alignment axis after the anterior chamber maintained by a cohesive viscosurgical device (Iviz, Bausch&Lomb). Finally, the surgery was uneventful after the viscosurgical device was removed manually using the balanced salt solution.

**Fig. 1:**
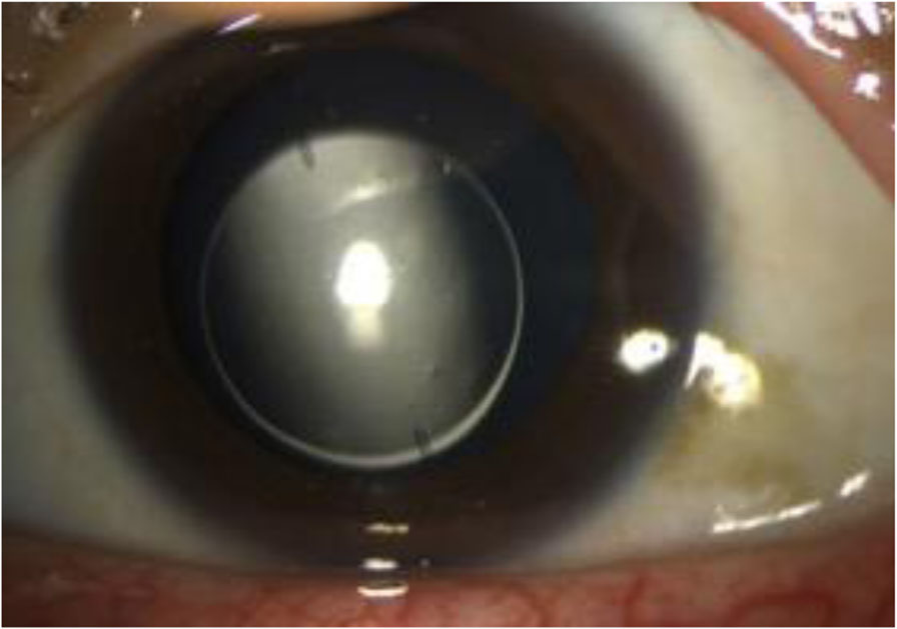
**a**. Slit-lamp image showing The TICL rotation of approximately 90° from its original position

On the first postoperative day, the left eye achieved an UDVA of 20/16 without any complications, while the vault was 360 μm. Twenty days after the surgery the patient presented urgently due to a sudden drop in visual acuity of his left eye when he woke up in the morning. He denied any trauma or other conditions. The UDVA decreased to 20/100 with a refraction of + 1.50–2.75 × 5°. After pupillary dilatation, a rotation of approximately 90° of the TICL from its original position was observed (Fig. 1). The vault was 336 μm. Thus, we decided to perform TICL reposition procedure in the operating room. The TICL remained stable with an UDVA of 20/16 and a vault of 365 μm until 1 month postoperatively, when the patient presented again with an exactly similar complaint. Conventional ophthalmic examination revealed almost the same angle and orientation of TICL rotation as the last time, while the vault was 342 μm.

After a careful and discreet re-evaluation, a replacement of the TICL with a larger size non-toric ICL was decided. A V4C (model: VICM12.6) with a power of − 11.5 D was chosen and placed in a horizontal position uneventfully. On 1 day postoperatively, the eye achieved an UDVA of 20/20 without any further complications, while the vault was 381 μm. During the next 2 months of follow-up, the patient’s condition has remained stable with an excellent post-operative outcome.

## Discussion and conclusion

A key factor in determining refractive outcome after TICL implantation is postoperative rotational stability. Packer et al. [4] reported that 0.33% (10/2970) of patients that underwent V4C implants required a second adjustment surgery and completely achieved the desired results subsequently. Similar occurrence rate (0.32%) was disclosed in retrospective analysis of 310 TICLs implantations in our refractive center since 2014 after one patient with a clear history of trauma was excluded. Theoretically, a rotation of 30° in the axis would result in a total loss of astigmatism correction [5]. The subject of the current report experienced spontaneous rotation of nearly 90° twice in the same eye. To the best of our knowledge, few studies have reported recurrent, significant, spontaneous rotation of a TICL.

TICL rotation has been reported to be associated with a very low postoperative TICL vaulting (<80 μm), [6] it is clear that a smaller TICL size will lead to a lower vaulting and more frequent rotation. Since the postoperative vault was in the normal range, we initially performed a TICL reposition, but unfortunately, rotation reoccurred 1 month postoperatively. Finally replacement for a larger size non-toric of ICL was performed. Traditionally, a lager vault responds to a larger size of ICL. However, in this patient the vault remained unchanged despite replacement with a larger ICL, this phenomenon reminded us the TICL rotation in this patient could not be simply explained by the vaulting and the size of the TICL.

**Fig. 2:**
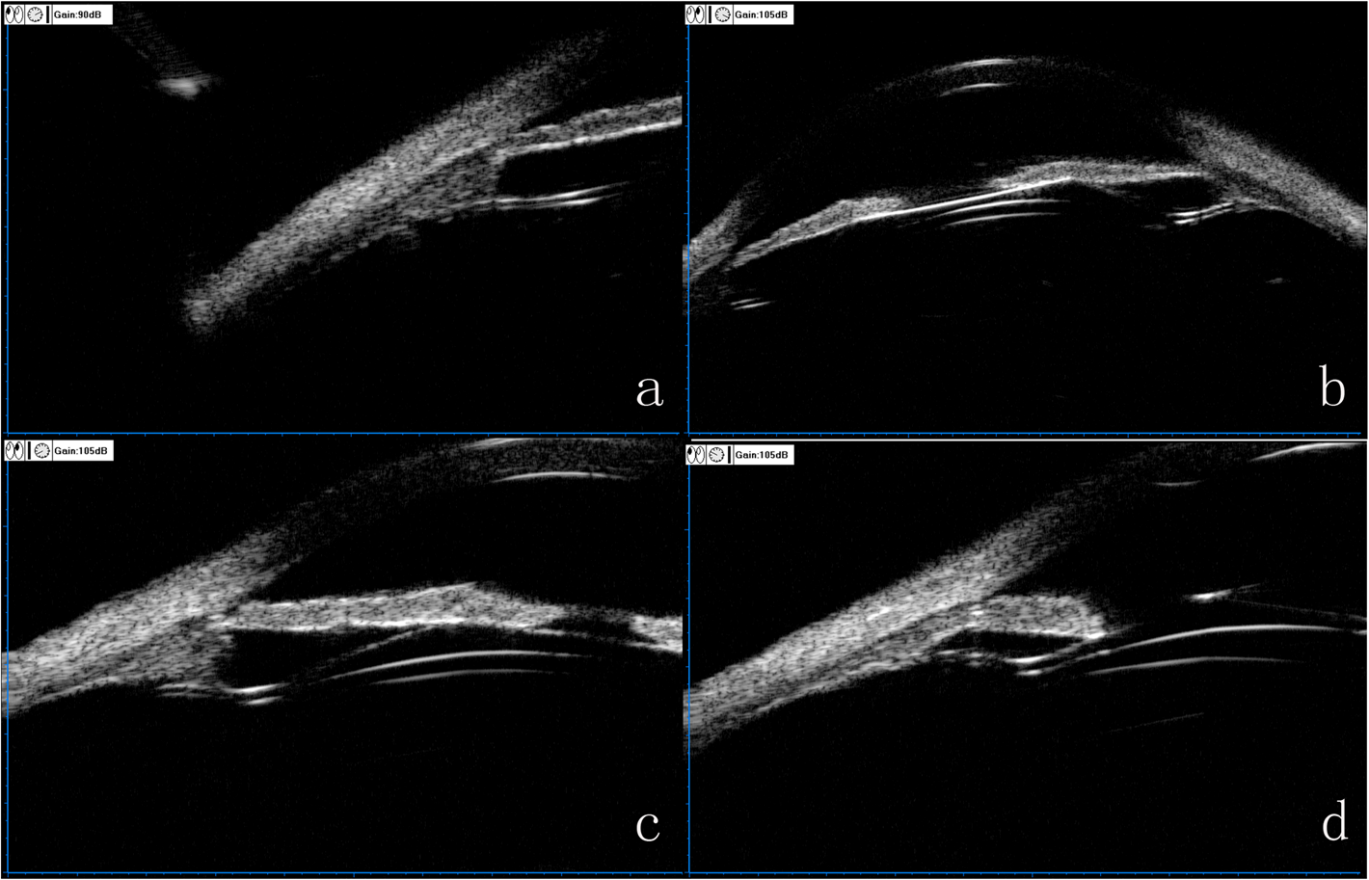
**a**-**d**. Ultrasound biomicroscopy image displaying the four footplates position, in which the footplate at 8 o’clock was located below the ciliary sulcus, while the other three footplates were inserted into the ciliary body

**Fig. 3:**
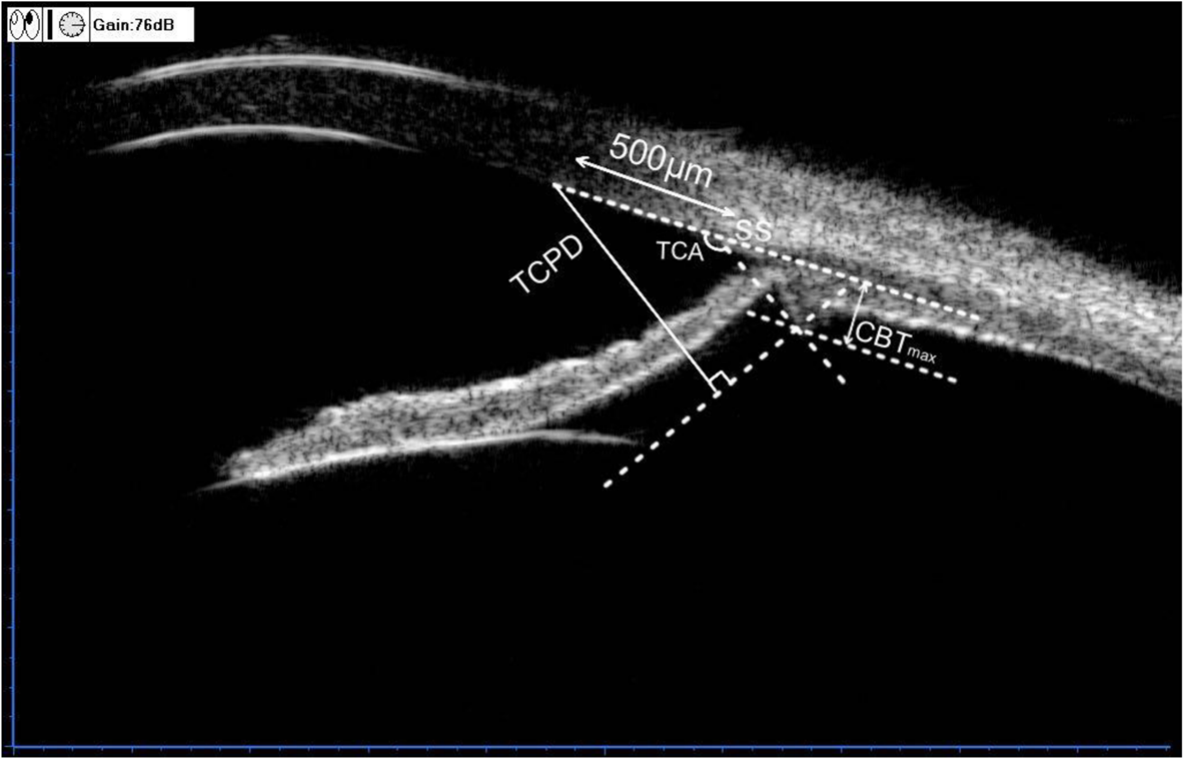
Assessment of the ciliary body parameters. Trabecular-ciliary process distance (TCPD) is the length of the line extending from the corneal endothelium 500 μm from the scleral spur perpendicularly to the line which passing through the most inner point of the ciliary body and parallel to the iris. Maximum ciliary body thickness (CBTmax) is the distance from the most inner point of the ciliary body to the inner wall of sclera or its extended line. Trabecular-ciliary angle (TCA) is the angle between the posterior corneal surface and the anterior surface of the ciliary body

Previously published work showed the ciliary sulcus measurement can improve the accuracy of the ICL sizing [7].The UBM imaging drawn our attention in which all the four footplates of ICL were constantly not in the ciliary sulcus postoperatively (Fig.2 a-d). Similarly, Park et al. [8] revealed a case with just one of four footplates located below the ciliary sulcus occurred a rotation of TICL with an angle of 11° after surgery. Zhang et al. [9] considered the main reason for the dislocation of the footplate was the surgical intrinsic drawback— blindly performed underneath the iris. We hypothesize the variation of ciliary sulcus structure is a main factor infecting the position of the footplates and the subsequent rotation of the TICL. Considering that the ciliary body was the major tissue composing the ciliary sulcus, we further analyzed the characteristics of the ciliary body on the UBM imaging and detected some unusual morphological features. In particular, the maximum ciliary body thickness (CBTmax), trabecular ciliary process distance (TCPD) and trabecular-ciliary angle (TCA) measured in this patient (Fig. 3) was 0.64 mm,1.43 mm and 140.2° respectively, significantly different from the mean values (1.053 ± 0.103 mm, 0.834 ± 0.234 mm and 75.1° ± 17.9°) reported in normal subjects [10, 11]. In summary, this patient had a posteriorly positioned ciliary body, a short and small ciliary process and a shallow ciliary sulcus. Part quadrant of the ciliary sulcus lost its normal length or exhibited a nearly obtuse angle. The “specious” ciliary sulcus leads the footplates to slide down to ciliary body easily or even cross over the small ciliary body coupled with changes in the accommodation states and finally rest on the lens periphery and zonules. When most footplates lost intrinsic support, a large rotation of the loosed TICL may occurred.

Close and long-term follow-up was warranted to observe if a recurrent rotation would happen in the future. Although the patient would not present with a sudden loss of visual acuity by the rotation of a ICL, the resulting impact on the lens and zonules still needed to be concerned.

This unique case revealed the potential association between the vault and instability of implanted ICL with the ciliary body morphology. Careful examination of UBM image can provide clinicans with valuable information in the preoperative ICL assessment. Further work including a large sample size with prospective design would be necessary to elucidate the actual mechanism.

## Data Availability

Not applicable.

## Abbreviations

CBTmax: Maximum ciliary body thickness
ICL: Implantable collamer lens
TCPD: Trabecular ciliary process distance
TCA: Trabecular-ciliary angle
TICL: Toric implantable collamer lens
UBM: Ultrasound biomicroscopy
UDVA: Uncorrected distance visual acuity

## References

[1] Hyun J, Lim DH, Eo DR (2017). A comparison of visual outcome and rotational stability of two types of toric implantable collamer lenses (TICL) : V4 versus V4c. PLoS One.

[2] Navas A, Munoz-Ocampo M, Graue-Hernandez EO (2010). Spontaneous rotation of a Toric implantable Collamer Lens. Case Rep Ophthalmol.

[3] Hashem AN, El Danasoury AM, Anwar HM (2009). Axis alignment and rotational stability after implantation of the toric implantable collamer lens for myopic astigmatism. J Refract Surg.

[4] Packer M (2018). The implantable Collamer Lens with a central port: review of the literature. Clin Ophthalmol.

[5] Ma JJ, Tseng SS (2008). Simple method for accurate alignment in toric phakic and aphakic intraocular lens implantation. J Cataract Refract Surg.

[6] Sheng XL, Rong WN, Jia Q (2012). Outcomes and possible risk factors associated with axis alignment and rotational stability after implantation of the Toric implantable collamer lens for high myopic astigmatism. Int J Ophthalmol.

[7] Dougherty PJ, Rivera RP, Schneider D (2011). Improving accuracy of phakic intraocular lens sizing using high-frequency ultrasound biomicroscopy. J Cataract Refract Surg.

[8] Park SC, Kwun YK, Chung ES (2009). Postoperative astigmatism and axis stability after implantation of the STAAR Toric implantable Collamer Lens. J Refract Surg.

[9] Zhang X, Chen X, Wang X (2018). Analysis of intraocular positions of posterior implantable collamer lens by full-scale ultrasound biomicroscopy. BMC Ophthalmol.

[10] Wang Z, Chung C, Lin J (2016). Quantitative measurements of the Ciliary body in eyes with acute primary-angle closure. Invest Ophthalmol Vis Sci.

[11] He N, Wu L, Qi M (2016). Comparison of Ciliary body anatomy between American Caucasians and ethnic Chinese using ultrasound biomicroscopy. Curr Eye Res.

